# Fluid Dynamics of Microgel-Covered Drops Reveal Impact on Interfacial Conditions

**DOI:** 10.3390/polym10080809

**Published:** 2018-07-24

**Authors:** Miriam Faulde, Eric Siemes, Dominik Wöll, Andreas Jupke

**Affiliations:** 1 Fluid Process Engineering, AVT–Aachener Verfahrenstechnik, RWTH Aachen University, Forckenbeckstrasse 51; D–52074 Aachen, Germany; miriam.faulde@avt.rwth-aachen.de; 2 Institute of Physical Chemistry–IPC, RWTH Aachen University, Landoltweg 2; D–52074 Aachen, Germany; siemes@pc.rwth-aachen.de (E.S.); woell@pc.rwth-aachen.de (D.W.)

**Keywords:** microgels, interfacial mobility, sedimentation, drop deformation, drag coefficient

## Abstract

Microgels are deformable polymer-networks with conspicuous properties. Their surface- activity associated with their switchability makes their application in liquid-liquid systems, such as extraction processes, particularly promising. For their application as switchable stabilizers at the interface, a detailed understanding of their impact on process relevant phenomena, such as the sedimentation behavior, is necessary. So far, the focus of research has been on microscopic-scale properties, whereby the propagation to macroscopic effects has rarely been quantified. In this study, single microgel-covered *n*-butyl acetate drops rising in a quiescent continuous water phase are investigated experimentally. The dependency of the microgel properties, in terms of size and cross-linking density, on the fluid dynamics are addressed. The impact of microgels is studied in detail by sedimentation velocity, drop deformation and the resulting drag coefficient. The deformation of drops is related to shape conserving interfacial properties such as the interfacial tension. Counter to our expectations, microgel-covered drops deform less than the drops of the pure system although microgels reduce the interfacial tension. Moreover, the sedimentation velocity is of special interest, since it reveals the mobility of the interface and friction conditions at the interface. Our results demonstrate the correlation between microgel properties at the interface on a microscopic scale and the macroscopic behavior of microgel-covered drops.

## 1. Introduction

Microgels are soft polymer particles which have gained significant interest over the last decades [1,2]. Microgels at liquid/liquid interfaces have been investigated on a microscopic level with regard to adsorption [3,4,5,6,7,8,9] and their assembly [3,4,5,6,7,9,10,11,12,13] connected to the microgels properties. Due to their soft and porous structure, their behavior at the interface is significantly different from that of rigid particles which are well-known stabilizers in “Pickering” emulsions. At liquid/liquid interfaces the lyophilic microgels deform and slightly protrude into the oil phase [10,12]. As they adsorb to the interface they reduce the interfacial tension [14] and change the rheological features like the elasticity of the interface [3,9].

First attempts towards establishing a relation between microgel properties and macroscopic effects are qualitative investigations on emulsion stability [3,5,12,15]. The emulsion stability was directly correlated to the microgels’ ability to deform at the interface [16]. More deformable microgels form more stable emulsions. The deformability is mainly governed by the cross-linker content [12,16], but also affected by the microgel size [6]. Weakly cross-linked microgels spread intensively at the interface and tend to form flat almost 2D networks with a significant overlap of entangling peripheraltical parts of the microgels [16]. As the cross-linker content increases, the microgels appear further separated with a clear protruding center [10,16]. The distance between the centers of the adsorbed microgels is not affected by their deformability [16]. Moreover, emulsion stability is also affected by the elasticity of the interfacial microgel layer, as the stability increases with increasing elasticity [3]. The detailed knowledge of their interfacial structure of microgels and the resulting macroscopic effects are essential for their application in extraction processes.

Due to their surface-activity, the application of microgels in liquid/liquid systems is promising, especially when stabilization can be switched [2,12]. Liquid/liquid systems are often limited by the contact area of the two liquid phases. To enlarge the interfacial area, one phase is dispersed into the other. Consequently, the efficiency of processes involving dispersed liquids is often limited by coalescence. In extraction processes for example, the separation of the value compound takes place across the liquid/liquid interface. Therefore, the mass transfer and, consequently, the separation efficiency are correlated to the drop size distribution in the extraction column. A promising approach to optimize the drop size is the utilization of microgels at the drop surface to stabilize the drop during the separation process. At the top of the extraction column, coalescence is required for phase separation, to obtain the value compound-rich organic phase. Utilizing microgels, the stabilization of the drops can be switched by a temperature shift to enable phase separation. Hence, regarding the application of microgels in disperse liquid/liquid systems, the behavior of mircogel-covered stabilized drops has to be investigated quantitatively on a macroscopic scale. Therefore, the interfacial conditions on the drop surface and their macroscopic effect on the fluid dynamics are in the focus of this study. The sedimentation velocity of drops is one of the relevant phenomena for the design of liquid/liquid contact apparatuses [17].

The sedimentation velocity can be derived by a force balance for the drop, comprising inertia, gravity, buoyancy and drag force. The drag force thereby accounts for the fluid dynamic forces resulting from shape and surface friction, revealing insights on the interfacial conditions.

The sedimentation behavior depends on the physical properties of the liquid/liquid system, such as density difference, viscosities, and interfacial tension [18], where the latter acts as shape conserving force towards spherical drops. The sedimentation velocity can be described as a function of the drop size and can be divided into three regimes [17,19,20,21]: small drops that behave like rigid particles, medium sized drops with internal circulation and large drops with oscillating shape. The transition drop size between these regimes depends on the solvent system [17,19].

**Figure 1:**
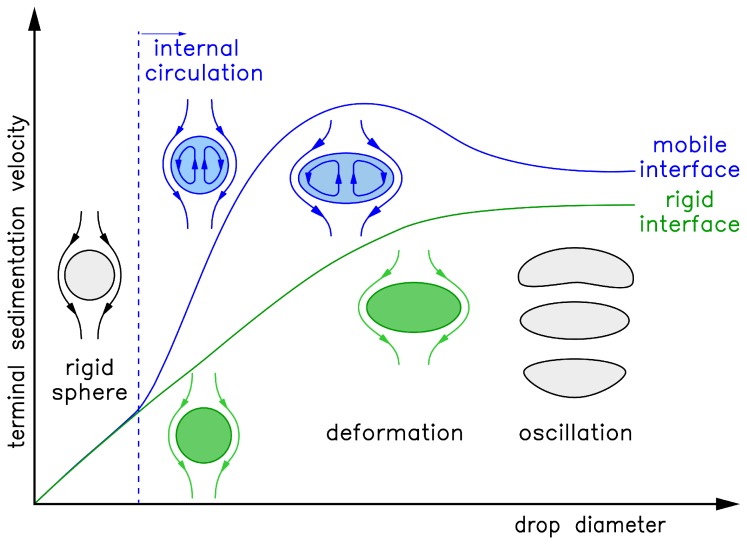
Fluid dynamics behavior of sedimenting single drops, drop shape and terminal drop velocity as a function of the drop diameter in case of a rigid interface (green line) and a mobile interface (blue line), the onset of internal circulation is indicated by the dotted blue line. (Based on [19])

Different terms of the force balance become more dominating depending on the drop diameter. The trend of the sedimentation velocity over drop diameter is illustrated in Figure 1. The velocity of small drops is preliminarily determined by the buoyancy force and therefore density difference of the liquid/liquid system. With increasing drop diameter the drag force becomes more relevant. For the interfacial conditions, two limiting cases are referred to: The mobile interface, where shear induced momentum transfer across the interface leads to internal circulation within the drop resulting in an acceleration, and the immobile or rigid interface (Figure 1 green line), which is characterized by an adhesion condition at the interface, leading to drops sedimenting like rigid fluid particles which also deform at larger diameters [17,18,19,21]. The sedimentation behavior of technical systems shows characteristics of both limiting cases (Figure 1 blue line) [19]. Small droplets sediment like rigid particles, due to impurity traces which accumulate at the interface and immobilize it. Since the interfacial area increases with increasing diameter, this effect decreases. At larger diameters, the friction at the interface of the rising drop induces a circulating flow within the drop, accelerating the drop. The onset of circulation is illustrated by the dotted blue line in Figure 1. In case of a rigid interface no momentum transfer and consequently no internal circulation occur, therefore no transition can be observed. The sedimentation velocity is maximal at diameters right before the onset of deformation. The drop deforms as the interfacial tension cannot preserve the spherical shape [19,20]. The onset of deformation is often correlated with a critical Weber number of We=4, which relates inertia force to interfacial tension as shape preserving force. The Weber number is defined as follows:

(1) $$We=\frac{u_{drop}^{2}d_{drop}\rho_{c}}{\gamma }$$

with drop velocity $u_{drop}$ [m/s], density ρ [$kg/m^{3}$], drop diameter $d_{drop}$ [m] and interfacial tension γ [N/m] [17,21]. Model approaches exist for the description of the sedimentation velocity as a function of the droplet diameter for the different regimes described above. The Henschke model combines theoretical models based on the Navier-Stokes equation and semi-empirical models for the different regimes to a single comprehensive model describing drops in all regimes from rigid sphere to oscillating regime. The model assumes the drop to be placed in a infinite Newtonian liquid under steady state conditions. The transition between the regimes and the corresponding models is fitted by transition parameters e.g., the parameter *d_um_* [19]. The parameter *d_um_* describes the diameter of the transition between the small drops acting like rigid spheres and the circulating drops (illustrated by the dotted blue line in Figure 1) [19,20]. For the limiting case of a rigid sphere, this parameter is infinite; for the case of an ideal mobile interface, it is set to zero.

Besides the velocity, the resulting drag force coefficient ($C_{d}$) is of interest for the fluid dynamic characteristic as described above. It can be determined experimentally via the drop size dependent sedimentation velocity described by [17]:

(2) $$u_{drop}=\sqrt[{2}]{\frac{4}{3}\frac{\Delta \rho }{\rho_{c}}gd_{drop}\frac{1}{C_{d}}}$$

For the prediction of drop velocity, many empirical correlations exist distinguishing between the interfacial conditions and considering the flow regime in terms of the Reynolds number. A detailed overview of these correlations is given by Wegener et al., 2014 [18].

## 2. Materials and Methods

### 2.1. Materials

In this study, water/*n*-butyl acetate is utilized as standard test system recommended by the european federation of chemical engineering (EFCE) [22]. Bidestilled water was utilized for the aqueous phase, for the organic phase *n*-butyl acetate of EMSURE^®^ quality from Merck (Darmstadt, Germany) was utilized. Prior to each experiment, the aqueous and the organic phase were mutually saturated, before microgels were added to the aqueous phase. As a reference surfactant CTAB (cetyltrimethylammonium bromide) from AppliChem (Darmstadt, Germany) is utilized. The critical micelle concentration (cmc) of CTAB is 0.8 mmol/L. All experiments involving the surfactant were performed at double cmc at 1.6 mmol/L.

### 2.2. Microgels

The utilized microgels were synthesized by aqueous, free radical precipitation polymerization [1] and purified by dialysis with a cellulose tube or by ultracentrifugation and dispersion in Milli-Q^®^ water. For the investigation of the effect of the microgel deformation at the interface, four different poly(n-isopropylacrylamide) (PNIPAM) microgels were synthesized with different radii and cross-linker content. Their characteristics are summarized in Table 1. The degree of cross-linking is defined by the utilized amount of cross-linker during synthesis and varies from 2.5 mol % to 20 mol %. The detailed composition of the reaction mixtures are listed in Table A2 in the Appendix A. The radius is determined by dynamic light scattering (DLS).

### 2.3. Sedimentation Velocity Measurement

**Figure 2:**
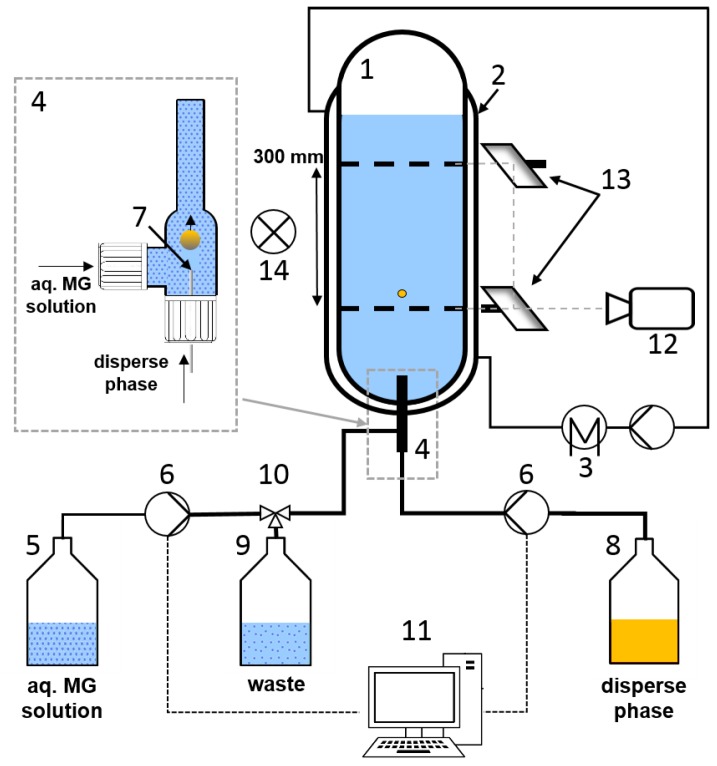
Experimental setup. (1) glass column, (2) jacket, (3) thermostat, (4) drop generator unit, (5) aq. microgel solution, (6) dosing system, (7) nozzle, (8) disperse phase, (9) waste, (10) three way valve, (11) computer control, (12) camera, (13) mirror, (14) illumination.

The experimental setup of the single drop cell as shown in Figure 2 is based on the standardized setup as described by [19,20,23]. The borosilicate glass cell (1) is 600 mm in height and has an inner diameter of 80 mm. The cell is equipped with a jacket (2) for temperature control. The temperature is adjusted to 25 ${}^{\circ }C$ using a thermostat (3, Julabo^®^ (Seelbach, Germany) MA4) which is connected to the jacket. To determine the terminal sedimentation velocity of single droplets, the time required to pass a defined length between two markings is taken with a camera (14) capturing 25 fps. To avoid perspective errors, the drop passing the second marking was observed through mirrors (13) that allow to view straight through the marked diameter of the cell. For better observation, the setup is illuminated using a LED panel (14). For the evaluation at least 5 drops are measured per drop diameter.

The evaluation is conducted by a frame-wise analysis of the videos. Two additional mirrors are installed to ensure that the drop has reached the terminal rise velocity when passing the measuring length. The length is thereby divided into three sections to detect unsteady velocities. The terminal drop velocity as a function of the drop diameter is fitted to the experimental data using the model approach from Henschke [19].

### 2.4. Generation of Microgel-Covered Drops

The experimental setup differs from the standard design regarding the drop formation. To enable the formation of microgel-covered drops and minimize the required amount of microgels, an new drop generator unit (4) was designed (Figure 2 close up). The drop generator inner diameter is 8 mm. Therefore, the drop diameter is limited to 6 mm to avoid wall contact. The generation of microgel-covered drops follows a three step procedure. First, 7 mL of aqueous phase with defined microgel concentration are filled from the vessel (5) to the drop generator via a Hamilton^®^ (Bonaduz, Switzerland) PSD2 dosing system (6) utilizing a 10 mL syringe. Then, the drop is formed at the tip of the nozzle (7) (nozzle diameter 0.3 mm, 1.25 mm and 1.8 mm are utilized depending on drop size), whereby the volume of the disperse phase (8) for the drop is dosed via a Hamilton^®^ PSD2 dosing system utilizing 2.5 mL and 5 mL syringes respectively. The drop rises through the drop generator and enters the cell where the measurement is conducted as described above. Subsequent to the measurement, the microgel solution is subducted from the drop generator and disposed to the waste (9) via the three way valve (10) to prevent the accumulation of microgels in the system and guarantee defined conditions during the drop generation. The drop size is defined by the liquid volume dosed by the pump. Consequently, drop diameters are indicated as volume equivalent sphere diameter.

Between the experiments, the set-up was cleaned using 10% sulfuric acid, acetone and bidestilled water.

### 2.5. Cryo-SEM Observation

The appearance of the interfacial microgel layer was observed by cryo-SEM (cryo-scanning electron microscope). The measurements are conducted utilizing a Hitachi S4800 FeSEM (Chiyoda, Japan) set-up. Prior to the measurement, an micro-emulsion is prepared, in order to derive interfaces in an observable scale. Per microgel type a 20 mL sample is prepared containing 6 mL *n*-butyl acetate and 14 mL aqueous microgel solution with a microgel concentration of 1 mg/mL. The micro-emulsion is prepared utilizing an IKA T 18 Ultra-Turrax^®^ (Staufen, Germany) stirring with 14.000 /min for 30 s. A sample of 10 μL is placed in the sample holder and frozen in liquid nitrogen. The sample is transferred to the microscope. Conditions in the microscope are kept to -140 ${}^{\circ }C$ and $4\times 10^{-5}$ mbar. In the preparation chamber, the sample is fractured with a scalpel-blade. Then the sample is sublimated twice for 90 s rising the temperature to -80 ${}^{\circ }C$. After decreasing the temperature again, the probe is sputtered with an Ag-Pd layer and transferred to the observation chamber.

## 3. Results and Discussion

### 3.1. Sedimentation Velocity

**Figure 3:**
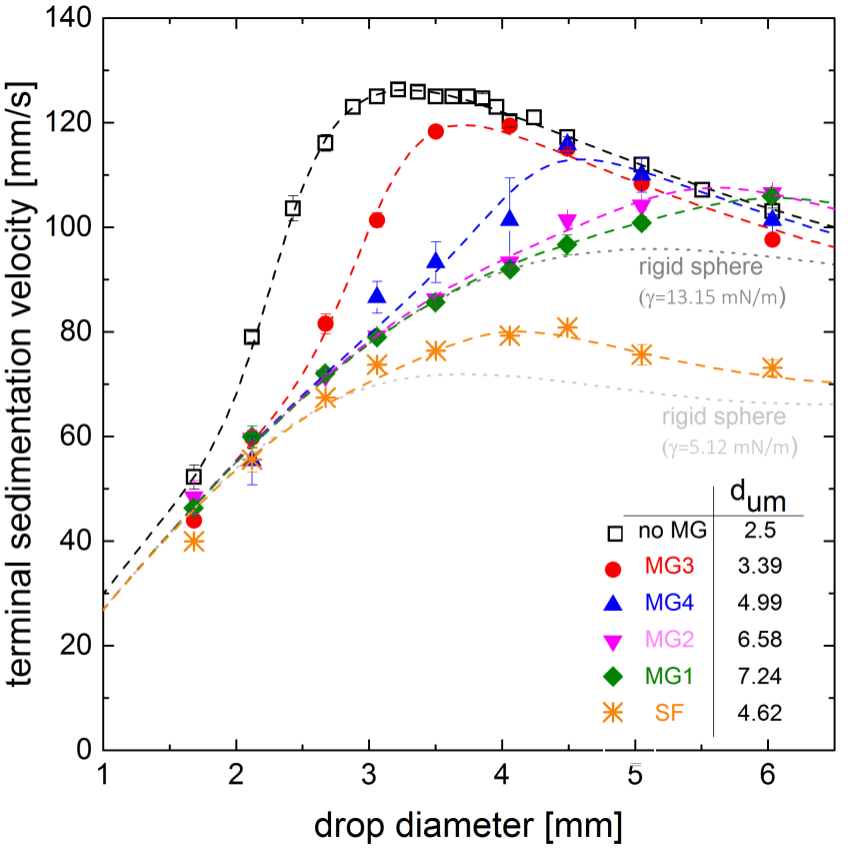
Terminal sedimentation velocity of *n*-butyl acetate drops in water, drops covered with different microgels (MG) and surfactant (SF) (microgel properties summarized in Table 1, $c_{MG}=0.047$ g/L in the drop generator for all microgels and $c_{SF}\gg cmc$), (□ pure system, ◆ MG1, ▼ MG2, ● MG3, ▲ MG4 and ∗ surfactant (SF), dashed lines obtained by fitting to Henschke Model [19,20], transition parameter *d_um_* listed in table)

Sedimentation velocity was measured in the single drop cell, as described above. Drop diameters between 1.68 mm and 6.03 mm were investigated, whereby the range is limited by the diameter of the drop generator. For reference, the sedimentation velocities of the pure water/*n*-butyl acetate system were measured (black squares in Figure 3). They are in good agreement with data obtained in previous studies [17,19,20]. The trend of the sedimentation velocity with increasing drop diameter shows a steep increase between drops of 1.68 mm and 3.22 mm from 52.22 mm/s to 126.26 mm/s, respectively. For drop diameters between 3.22 mm and 4.06 mm, the velocity is almost constant. For diameters larger than 4.06 mm, the velocity decreases. In the experiment, we observed a deformation of these drops. The deformation increases with increasing drop diameters. For diameters larger than approximately 4 mm, oscillation was observed. The deformation is discussed in more detail in Section 3.2. The biggest drops observed of 6 mm diameter rise with about 100 mm/s. This trend in sedimentation velocity for increasing drop diameters is identifying for systems with mobile interfaces. For the investigated system, the circulating regime, which is characterized by acceleration due to the onset of internal circulation, starts between drop diameters of 2 mm and 3 mm, which is also reflected by the transition parameter *d_um_* of the Henschke model [19].

For the investigation of the impact of microgels at the interface and their degree of cross-linking, the sedimentation velocities of MG1-, MG2- and MG3-covered drops are compared. The corresponding degrees of cross-linking are 2.5 mol %, 5 mol % and 20 mol %, respectively. The degree of cross-linking is directly related to the spreading of the microgels at the interface [5,12]. The drop diameter does not affect the spreading of the microgels since the drop radius is on a millimeter-scale causing no significant curvature of the interface on micro-scale. The microgels MG1, MG2 and MG3 have almost the same diameter, but MG3 has the highest investigated degree of cross-linking and consequently it spreads less at the interface and interpenetration between the adjoining microgels is less than for the weaker cross-linked microgels MG1 and MG2. The experimental results and the fitted curve of the Henschke model [19] are shown in Figure 3. The obtained sedimentation velocities for drop diameters between 1.68 mm and 4.06 mm differ significantly from the pure system data. The largest difference can be found for 2.46 mm drops with velocities of 81 mm/s and 101 mm/s, respectively. This indicates the impact of the microgel within the circulating regime. The results for MG1- and MG2-covered drops are different from the results obtained from MG3-covered drops. Up to a drop diameter of about 5 mm, the sedimentation velocity increases with an almost constant trend. The values also match the limiting case of a rigid sphere for diameters up to 4 mm. At larger diameters, the MG1- and MG2-covered drops rise more quickly than the limiting case, whereby the velocity of the weaker cross-linked MG1-covered drops is slightly slower compared to MG2-covered drops. However, for both microgels no clear maximum in sedimentation velocity can be detected in the observed drop diameter range. This indicates the absence of drop deformation and oscillation for that range. The less profound dependency between the sedimentation velocity and the drop diameter implies that the velocity increases solely by the increasing buoyancy force with increasing drop diameter and that the microgels prevent the circulation. Moreover, during the experiments no significant deformation of the droplets covered with these microgels was observed, thus matching the absence of a maximum. However, the displayed limiting case of the pure system does not consider the reduced interfacial tension.

Comparing the microgels with respect to their degree of cross-linking, it can be summarized that the impact on the sedimentation velocity increases with decreasing cross-linker content, as regards the maximum velocity as well as the trend of the velocity over the drop diameter. This is especially apparent regarding the dependency between the onset of the acceleration and the degree of cross-linking of the utilized microgels. The drops covered with the highest cross-linked microgels accelerate at larger diameters than the drops of the pure system, while there is no observable acceleration for the less cross-linked microgels MG1 and MG2. An explanation for the observed behavior is a shifted onset of internal circulation to larger diameters for the intensely cross-linked microgel MG3 and a suppression of internal circulation for microgels MG1 and MG2. This is also reflected in the development of the transition parameter *d_um_* in the Henschke model [19]: as the cross-linker content is increased, *d_um_* is reduced. A linear correlation between the transition parameter *d_um_* and the degree of cross linking for MG1 to MG3 is found (compare table in Figure 3). Since the degree of cross-linking predominately affects the interpenetration of the adjoining microgels [6,9], which is difficult to measure, the quantitative interpretation of the effect is not feasible. Nevertheless, it can be concluded, that a strongly interpenetrated network at the interface, as it is formed by weakly cross-linked microgels like MG1 and MG2, inhibits the momentum transfer across the interface more drastically compared to a layer of highly cross-linked microgels, like MG3, which are less interconnected. The other aspect is the shift of the maximum of velocity to larger diameters utilizing weaker cross-linked microgels; this could be an explanation to an increased stabilization of the droplets resulting in a delayed onset of deformation at larger diameters which will be discussed in Section 3.2. The impact of microgel size on sedimentation velocity can be determined comparing microgels MG2 and MG4, with a radius measured in bulk of 287 nm and 170 nm, respectively. For small drop diameters up to 2.5 mm, both show the same trend. For larger drop diameters, the velocity of the drops covered with the smaller microgel MG4 is faster. The maximum can be determined at 4.5 mm with 115 mm/s. For further increasing diameters the velocity decreases as the drop deforms and is slower than the less deformed MG2-covered droplets. Similar to the effect of the degree of cross-linking, it can be concluded that larger microgels affect sedimentation velocity, and thus the interfacial mobility, more strongly as they spread more intensely at the interface and form a barrier to momentum transfer inhibiting the formation of internal circulation.

**Figure 4:**
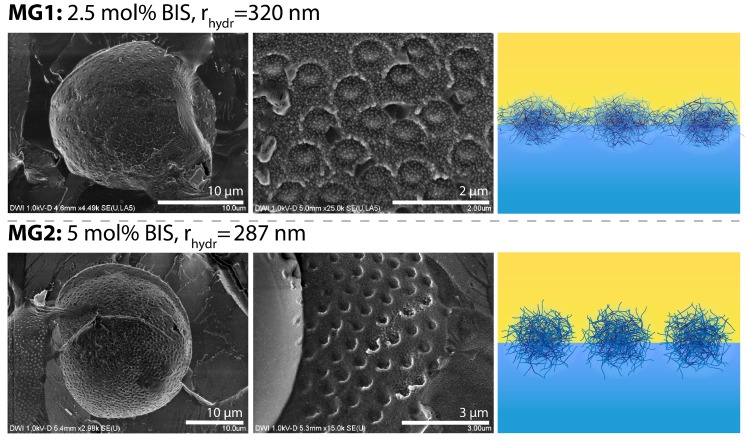
Cryo-SEM images of microgel-covered *n*-butyl acetate drops with different degree of cross-linking (**left side** and **middle**) and illustration of the microgel arrangement at the interface (**right side**).

Relating these observations to the microgel properties, it can be concluded that the impact of the microgels on the sedimentation velocity and deformation increases with decreasing degree of cross-linking whereas for constant cross-linking the impact increases with increasing size. Figure 4 shows images from cryo-SEM observation of the utilized microgels at the interface. The weakest cross-linked microgel MG1 appears almost as a smooth film. However, the individual microgels are still recognizable. In contrast, the medium cross-linked microgel MG2 appears to be more bumpy at the interface. Since the cross-linking and the size affect the microgel deformability at the interface and the magnitude of the effects on the sedimentation velocity also depend on the degree of cross-linking and the size, these findings allow for a correlation of the effects to the ability of the microgel to deform at the interface. The larger and less cross-linked microgels spread more at the interface, resulting in a stronger barrier for impulse transfer. This hypothesis is encouraged by the cryo-SEM observation displayed in Figure 4. Additionally, due to the different plasticity of the microgel-covered interface, a fluid mechanical impact in terms of friction behavior needs to be further investigated, since the texture of a surface at a nano- or microscopic scale can significantly affect the hydrodynamic flow patterns [24].

**Figure 5:**
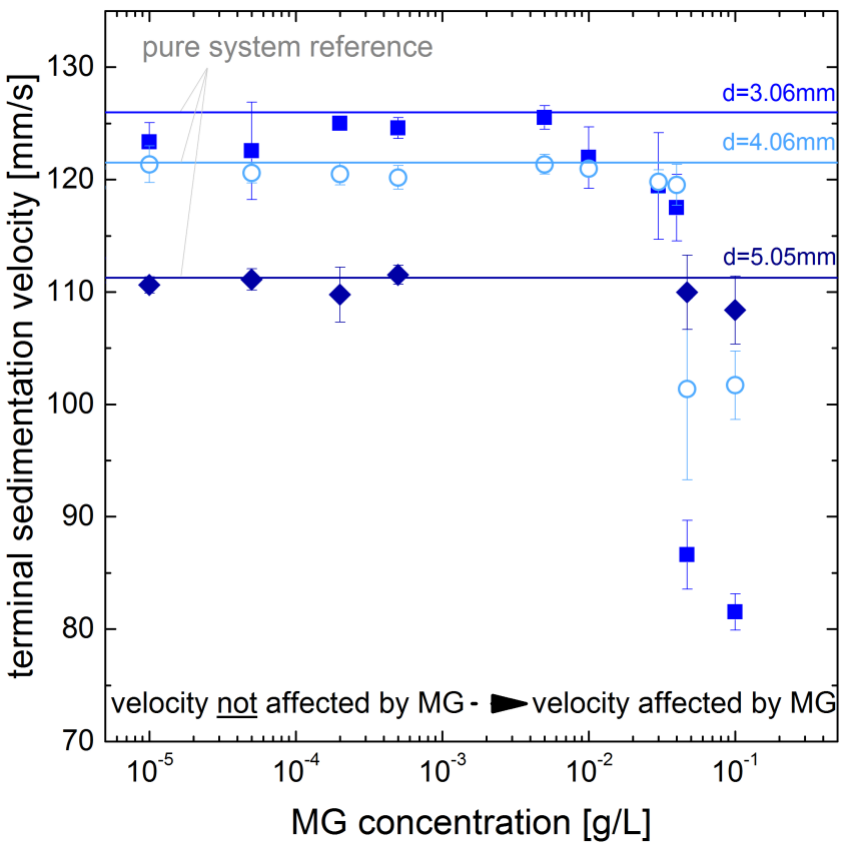
Impact of microgel concentration of MG4 in the drop generator on sedimentation velocity for different drop diameters (lines indicate pure system reference).

The impact of the microgel concentration in the drop generator is shown exemplary for the microgel MG4 and drop diameters of 3.06 mm, 4.06 mm and 5.05 mm (see Figure 5). The drop generator is designed to allow a defined coverage of the drops. As the microgel solution within the drop generator is replaced for each drop to avoid dilution effects, the amount of microgels available for adsorption on the drop surface ($n_{MG}$) can be calculated from the microgel concentration in the drop generator ($c_{MG}$) and the volume of the path of the rising drop within the drop generator ($V_{path}$):

(3) $$n_{MG}=c_{MG}\;\cdot \;V_{path}\;\;\;\mathrm{with}\;\;\;V_{path}=\pi \frac{d_{drop}^{2}}{4}\;\cdot \;h_{path}$$

The number concentration of the microgels is determined by the formula introduced by Destribates et al., 2014 [6]. Since the drop is generated at a nozzle tip above the bottom of the generator, the height of the path is less than the height of the drop generator.

For low microgel concentrations in the drop generator, the obtained velocities match the pure system velocities (Figure 5 horizontal lines). When increasing the concentration to 0.047 mg/mL, an abrupt reduction in velocity for 3.06 mm and 4.06 mm drops is observed. Therefore, it is assumed that drops are fully covered when leaving the drop generator with concentrations above 0.047 mg/mL. To rationalize this result, we estimated that the drop can be fully covered with microgels at this concentration. Assuming the area which a single microgel covers at the interface as the circular area of its diameter measured in bulk and that all microgels within the path described by Equation (3) adsorb to the drop surface, we obtain an area covered by the microgels approx. 100 times larger than the surface of the drop. Thus, the concentration of microgels in the path is by far sufficient to cover the full drop at a concentration of 0.047 mg/mL.

### 3.2. Drag Coefficient and Deformation of Drops

**Figure 6:**
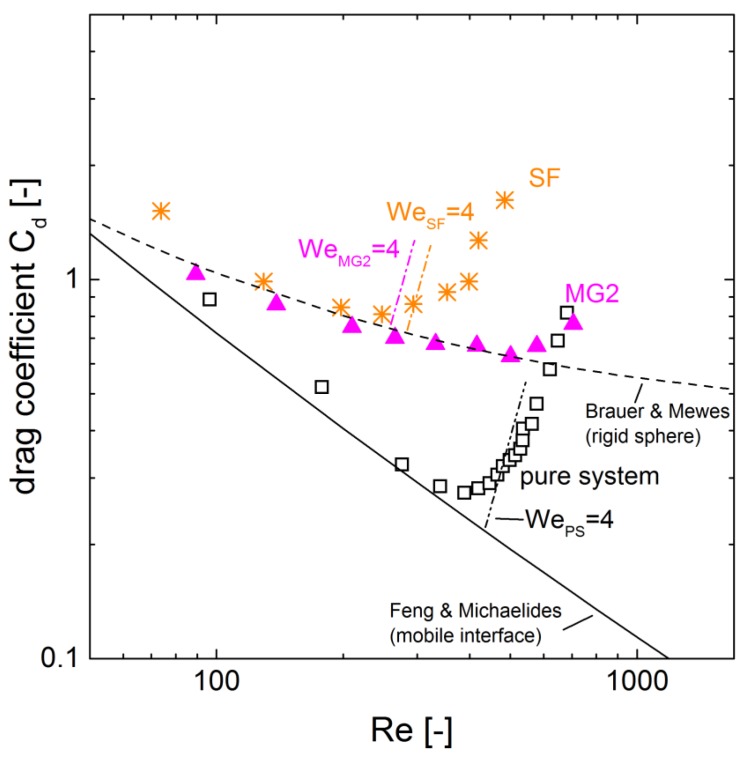
Drag coefficient as a function of the Reynolds number for the pure water/*n*-butyl acetate system and the system containing MG2 and surfactant, respectively. The shown Weber number indicates the predicted onset of drop deformation. The development of the Weber number with increasing drop diameter is shown in more detail in Figure 7.

The shift of the maximum velocity to larger drop diameters for microgel-covered drops in Figure 3 already indicates an effect on the drop deformation. This effect becomes more obvious regarding the drag of the droplets. The drag coefficient is derived from the drop diameter and the velocity [17,19]. In Figure 6 the drag is displayed as a function of the Reynolds number. The trend of rigid spheres and that of mobile interfaces are determined by the empirical models from Brauer and Mewes [25], and Feng and Michaelides [26], respectively.

The pure system is in good agreement with the trend of the mobile interface from Feng et al. [26] for Reynolds numbers smaller than 300. The drag coefficient increases as the droplet begins to deform for larger Reynolds numbers. The onset of deformation is often related to the Weber number (Equation (1)), which rates the fictitious force of the drop to the stabilizing surface force. The deformation starts for We≥4 [17,27]. For the pure system, this approach matches the findings very well (Figure 3 and Figure 6).

For the surfactant-covered drops, the drag coefficient is larger than for the pure system and for the microgel-covered drops. For Reynolds numbers smaller than 250 , the decreasing trend of the drag coefficient is similar to the model of a rigid sphere. The increase in the drag coefficient at Reynolds number 260 is in good agreement with the critical Weber number. However, the drag increases less with increasing Reynolds number than for the pure system.

For the microgel-covered drops, the drag coefficient is significantly larger than for the pure system and can be described by the trend of the rigid sphere model from Brauer and Mewes [25] for Reynolds numbers smaller than 500. For larger Reynolds numbers, the drag coefficient increases, but this increase is less pronounced than for the pure system. The increase estimated by the critical Weber number is at Reynolds number 260. The prediction of the onset of deformation by the critical Weber number does not hold for the microgel-covered drops.

**Figure 7:**
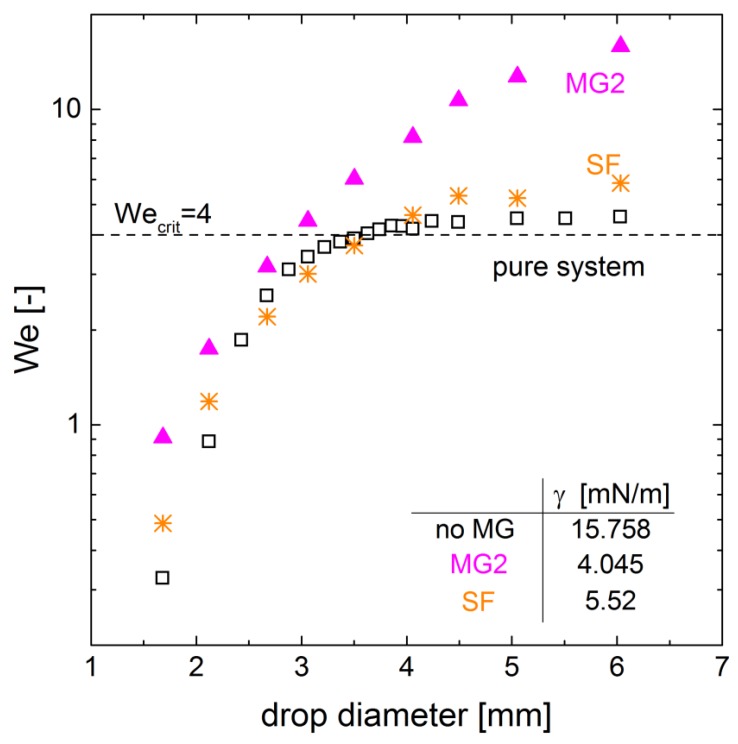
Weber number as a function of the drop diameter, for the pure water/*n*-butyl acetate system and the system containing MG2 and surfactant, respectively. The critical Weber number We=4 refers to the onset of deformation [18,21]. The interfacial tensions of the three different systems applied in the Weber number are given in the table (standard deviation below $1.8\times 10^{-2}$ mN/m).

**Figure A1:**
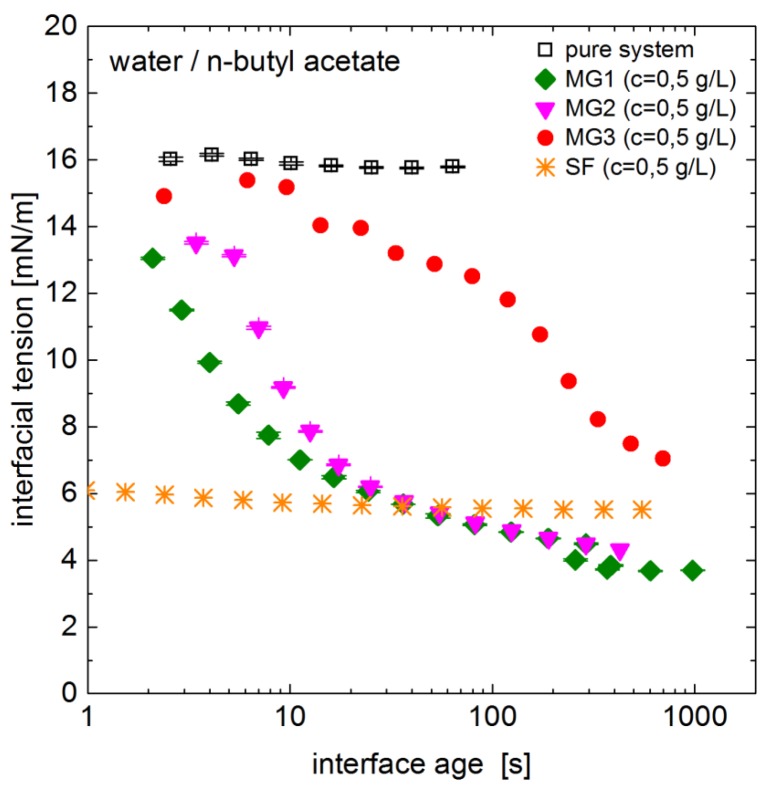
Interfacial tension of the water/*n*-butyl acetate system with different microgels and surfactant, measured by drop volume tensiometry with DVT50 from Krüss (Hamburg, Germany).

The development of the Weber number over drop diameter is shown in Figure 7. The Weber number increases with increasing diameter and velocity. For the pure system the Weber number reaches a plateau after the value exceeds the critical value of *We* = 4. The trend for the surfactant system is comparable. The surfactant reduces the interfacial tension significantly but the sedimentation velocity is also reduced leading to a similar trend for the Weber number over the drop diameter as for the pure system. A comparison of the diameter of the maximum velocity in Figure 3 and the diameter of the critical Weber number in Figure 7 shows only a small deviation for the pure and the surfactant system, respectively. However, the characteristics of the deformation of microgel-covered drop are not correctly displayed. The critical Weber number is reached for drops about 3 mm, but there is no maximum in velocity observable in this drop diameter range (compare Figure 3). This discrepancy can be explained by the definition of the Weber number (Equation (1)), where the shape preserving force is solely accounted for by the interfacial tension. The measured values for the interfacial tension are listed in Figure 7, and the complete data of the dynamic interfacial tension measurement is shown in Figure A1 in the Appendix A. Since the microgels reduce the interfacial tension the most, the obtained Weber numbers are larger, and the critical Weber number is reached for smaller diameters and slower velocities, respectively. Since the microgel layer at the interface does not solely reduce the interfacial tension but also affects the mechanical properties such as the viscoelasticity [3,9,28], more detailed approaches are required for the the adequate description of this behavior.

**Figure 8:**
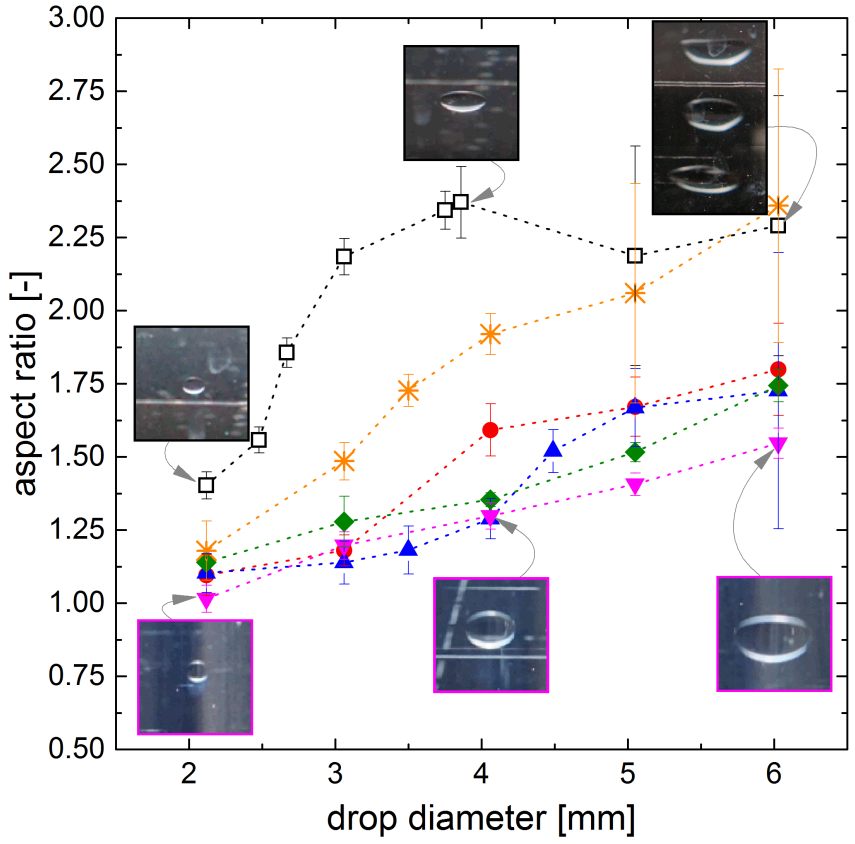
Deformation of drops displayed as aspect ratio (width to height ratio) ((□ pure system, ◆ MG1, ▼ MG2, ● MG3, ▲ MG4 and ∗ surfactant (SF)). Microgel-covered drops generated with 0.047 mg/mL, surfactant data for $c_{SF}\gg cmc$, dotted lines as guide to the eyes.

Besides the analysis of dimensionless quantities, the deformation of the drops was also investigated directly by the determination of the aspect ratio of the drops. The results are shown in Figure 8. The deformation of the pure system is most pronounced. For drop diameters larger than 3 mm, the aspect ratio remains constant but the error bars increase drastically due to increased shape oscillation of the drops. The drops of the surfactant system show a deformation at larger diameters, although the reduced interfacial tension would favor an earlier onset of the deformation. The delayed onset can be explained by viscoelastic forces acting in indirect proportion to the reduced interfacial tension as demonstrated by Paul 2014 [29]. The microgel-covered drops show significantly lower deformation compared to the pure system and the surfactant system. With increasing drop diameter, the deformation increases slightly and becomes almost linear. There are no clear differences in this trend for the different microgels observable. Regarding the elastic properties of interfacial microgel layers, it is known that these are predominantly affected by the polymer type and the packing density of the layer. The cross-linking does not significantly affect the mechanical properties of the interfacial microgel layer [9]. This matches the absence of differences for the utilized micorgels, since all utilized microgels are pure PNIPAM microgels, and their packing density at the interface is similar due to the equal conditions in the drop generator as observed for the microgels MG1 and MG2 (Figure 4).

**Figure 9:**
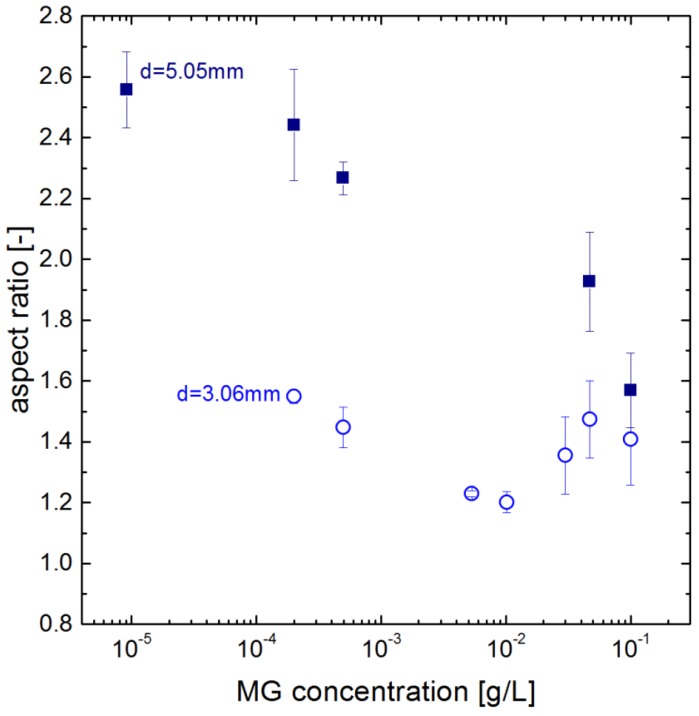
Dependency of the deformation (aspect ratio of width and height) of drops covered with MG4 on microgel concentration in the drop generator.

The impact of the microgel concentration on the deformation is also considered and shown in Figure 9. At smaller diameters, e.g., for 3.06 mm, the deformation does not show a clear trend. For the larger drops, shown for 5.05 mm in Figure 9, the deformation decreases with increasing concentration in the drop generator. This supports the assumption that the drops reach a full coverage at higher concentration in the drop generator. Regarding the relation of the deformation to mechanical properties discussed above, this indicates the impact of the packing density.

The other finding is that a reduced deformation and therefore a more spherical drop shape would result in a reduced drag and consequently in a faster sedimentation velocity. However, the opposite effect is observed, as the sedimentation velocity of the more spherical drops is diminished. This could be explained by a suppression of internal circulation as the microgel-coverage of the drops interface increases. An other possible explanation would be an increase in drag due to the interfacial roughness implied by the microgels.

## 4. Conclusions

In this study we investigated the impact of microgels on the fluid dynamics of single drops with regard to the application of microgels in liquid/liquid processes such as extraction. We demonstrated a correlation between the fluid dynamic behavior of microgel-covered drops and the properties of the microgels. Compared to the pure system, the sedimentation velocity is more reduced and the maximum of velocity is shifted to larger drop diameters with increasing spreading of the microgels at the interface.

Furthermore, the microgels make the drops interface more resistant against deformation although they reduce the interfacial tension. This can be explained by the mechanical properties of the microgel layer. Therefore, the common tools for the prediction and description of the fluid dynamics like dimensionless quantities cannot be applied as they consider solely the interfacial tension as shape conserving force.

The reduced sedimentation velocity at simultaneous decreased deformation of the drops indicate the large impact of the interfacial microgel layer. The origin of the effect has to be determined by more detailed analysis of the interfacial conditions. The contribution of friction conditions and drop internal flow has to be determined. A suppression of internal circulation within the droplets by the microgels would also effect mass transfer in a disperse system as the circulation leads to a better mixing and therefore to a decrease in concentration gradient within the drop, making these phenomena especially interesting regarding application processes.

## Figures and Tables

**Table 1:** Cross-linker content and hydrodynamic radius of the microgels.

Name	Cross-Linker Content	Hydrodynamic Radius
**-**	**[mol %]**	**[nm]**
MG1	2.5	320
MG2	5	287
MG3	20	310
MG4	5	170

## Abbreviations

The following abbreviations are used in this manuscript:

MDPI	Multidisciplinary Digital Publishing Institute
BIS	N,N-Methylenebis(acrylamide)
cmc	critical micelle concentration
CTAB	cetyltrimethylammonium bromide
DLS	dynamic light scattering
EFCE	European Federation of Chemical Engineering
MG	microgel
SEM	scanning electron microscope
SF	surfactant
PNIPAM	Poly(*N*-isopropylacrylamide)

## Appendix A

**Table A1:** List of symbols.

Symbol		Unit
$C_{d}$	drag coefficient	-
$c_{MG}$	microgel concentration	mol/m^3^
$d_{drop}$	drop diameter	m
$d_{um}$	transition parameter	mm
$h_{path}$	height of the drop path in drop generator	m
$n_{MG}$	number of mcirogels	-
$r_{hydr}$	hydrodynamic radius	m
$u_{drop}$	drop velocity	m/s
$V_{path}$	Volume of the drop path	m
dimensionless numbers		
We	Weber number	-
Re	Reynolds number	
greek letters		
γ	interfacial tension	N/m
$\rho_{c}$	density of the continuous phase	kg/$m^{3}$
$\rho_{c}$	density of the disperse phase	kg/$m^{3}$
Δρ	density difference	kg/$m^{3}$

**Table A2:** Microgel synthesis parameters.

Component	Unit	MG1	MG2	MG3	MG4
NIPAM	g	5.4008			4.5443
BIS	g	0.18303	0.3677	1.4708	0.3329
water	mL	450			172
AMPA	g	0.1503			
APMH	g				0.1228
V50	g				0.0468
CTAB	g				0.0058
reaction conditions					
stirring speed	rpm	200			500
reaction time	h	2			3.5
Temperature	${}^{\circ }C$	70			67
purification			cellulose dialysis		ultracentrifugation
